# 12 Weeks of Kindergarten-Based Yoga Practice Increases Visual Attention, Visual-Motor Precision and Decreases Behavior of Inattention and Hyperactivity in 5-Year-Old Children

**DOI:** 10.3389/fpsyg.2019.00796

**Published:** 2019-04-10

**Authors:** Sana Jarraya, Matthias Wagner, Mohamed Jarraya, Florian A. Engel

**Affiliations:** ^1^Research Unit, High Institute of Sport and Physical Education, University of Sfax, Sfax, Tunisia; ^2^Department of Sport Science, Bundeswehr University Munich, Neubiberg, Germany; ^3^Department Movement and Training Science, Institute of Sport and Sport Science, Heidelberg University, Heidelberg, Germany

**Keywords:** behavior modification, cognition, executive functions, preschool, exercise intervention

## Abstract

The present study assesses the impact of Kindergarten-based yoga on cognitive performance, visual-motor coordination, and behavior of inattention and hyperactivity in 5-year-old children. In this randomized controlled trial, 45 children (28 female; 17 male; 5.2 ± 0.4 years) participated. Over 12 weeks, 15 children performed Hatha-yoga twice a week for 30 min, another 15 children performed generic physical education (PE) twice a week for 30 min, and 15 children performed no kind of physical activities, serving as control group (CG). Prior to (*T*_0_) and after 12 weeks (*T*_1_), all participants completed Visual Attention and Visuomotor Precision subtests of Neuropsychological Evaluation Battery and teachers evaluated children’s behavior of inattention and hyperactivity with the Attention-Deficit/Hyperactivity Disorder (ADHD) Rating Scale-IV. At *T*_0_, no significant differences between groups appeared. Repeated measures analysis of variance revealed that following Bonferroni–Holm corrections yoga, in comparison to PE and CG, had a significant positive impact on the development on behavior of inattention and hyperactivity. Further, yoga has a significant positive impact on completion times in two visumotor precision tasks in comparison to PE. Finally, results indicate a significant positive effect of yoga on visual attention scores in comparison to CG. 12 weeks of Kindergarten-based yoga improves selected visual attention and visual-motor precision parameters and decreases behavior of inattention and hyperactivity in 5-year-old children. Consequently, yoga represents a sufficient and cost-benefit effective exercise which could enhance cognitive and behavioral factors relevant for learning and academic achievement among young children.

## Introduction

As an important basis for academic achievement in children and adolescents, adequate cognitive functions are suggested (Duncan et al., 2007; McClelland et al., 2013). Whereas, cognitive functions are defined as an individual’s mental processes, including memory, attention, learning, problem solving, reasoning, and decision-making (Matlin, 2009).

Recently, a growing body of literature appears examining the effects of acute and chronic physical activity in young and preadolescent children and its impacts on cognitive and executive functions as well as on academic achievement (Verburgh et al., 2014; Ludyga et al., 2016; de Greeff et al., 2018; Singh et al., 2018). Whereas, the association between executive functions and academic achievement is consistently demonstrated and emphasized (Blair and Raver, 2015). The concept of executive functions comprises several cognitive functions and refers to at least three interrelated cognitive functions (Miyake et al., 2000): inhibition, shifting, and updating (for overview, see Diamond, 2013). In this context, recent research demonstrated that physical activity can lead to improved cognitive and executive functions and enhanced academic achievement in young students (Sattelmair and Ratey, 2009; Fedewa and Ahn, 2011; Verburgh et al., 2014; de Greeff et al., 2018; Oberer et al., 2018). More precisely, a recent meta-analysis showed that physical activity has a small positive effect on school engagement in students aged 6–15 years (Owen et al., 2016). Since school engagement is a crucial factor determining academic performance (Perry et al., 2010), this demonstrates the potential of physical activity for academic achievement. Furthermore, reviewed data suggest that in young preadolescent students (6–12 years), acute physical activity has a positive effect on attention, while chronic physical activity reveals a positive effect on executive functions, attention, and academic performance (de Greeff et al., 2018). In this context, Alesi et al. (2014) demonstrated that, as a result of regular Karate practice, 9-year-old Karate athletes exhibited better working memory and visual selective attention compared to their sedentary peers.

The impact of physical exercise on deficits of attention in children is a related subject and of relevance to the present study, because disturbances revealing a deficit of attention are displayed as one of the reasons of academic failure as much as they interfere with requirements of the school (Flessas and Lussier, 2001). Rates of Attention-Deficit/Hyperactivity Disorder (ADHD) in preschool children are rising constantly and ADHD prevalence is estimated to be at 2.1% among U.S. American preschool children (Danielson et al., 2018). ADHD is a neurodevelopmental disorder characterized by symptoms of inattention and hyperactivity/impulsivity, which potentially causes lifetime impairment for afflicted individuals (American Psychiatric Association [APA], 2013). Since attention is a cognitive function and strongly implicated in the processes of learning (Flessas and Lussier, 2001) for both simple and complex activities (Flessas and Lussier, 2001; Delvolvé, 2005), we intended to analyze how a sensory-motor training in children influences behavior of inattention and hyperactivity and cognitive performance.

In this context, numerous studies demonstrated the significant effects of a sensory-motor training in children in terms of (i) decreased hyperactivity, anxiety, depression, and aggression (Banaschewski et al., 2001; Rosen et al., 2015) and (ii) reduction of behavioral and emotional problems (Sibley and Etnier, 2003; Haapala, 2012; Singh et al., 2012; Lees and Hopkins, 2013; MacCobb et al., 2014; Sandford et al., 2015). Consequently, physical activities and appropriate training programs are likely to boost cognitive functions (Hillman et al., 2014; Schmidt et al., 2015; Altenburg et al., 2016) and improve behavior and mood (Delvolvé, 2005; Gothe et al., 2013; MacCobb et al., 2014), whereby younger children seem to reveal higher benefits (Leconte, 2005).

Yoga is suggested to be an effective sensory-motor training for children to reduce behavioral and emotional problems and improve cognitive functions which might have a positive impact on academic performance (Chaya et al., 2012; Chou and Huang, 2017; Bazzano et al., 2018; Cohen et al., 2018). Recent studies demonstrated that yoga is one intervention with the potential to increase attention and academic performance in children (Chaya et al., 2012; Khalsa et al., 2012; Noggle et al., 2012; Chou and Huang, 2017) and adolescents (Khalsa et al., 2012; Noggle et al., 2012; Purohit and Pradhan, 2016). Yoga practice includes stretching postures, breathing exercises, and meditation (Taimini, 2005; Brethenoux-Seguin, 2007; Rosen et al., 2015). Indeed, conscious breathing has an impact on the parasympathetic system, causing a relaxation (Innes et al., 2005). Since yoga is a conscious exercise that builds attention control and inhibitory skills (Kaunhoven and Dorjee, 2017), it may have the potential to increase attention span on task during class and may represent an enjoyable and cost-effective treatment for children with and without attention problems (Peck et al., 2005).

Indeed, a growing body of evidence evolved underpinning the potential of yoga to support cognitive functioning in children and adolescents in different settings. Yoga was effective in improving delayed recall of spatial information and verbal memory in 11–16-year-old children (Manjunath and Telles, 2004). Similar results were reported for three months of yoga practice on executive functions of adolescent dwelling in an orphan home (Purohit and Pradhan, 2016). Yoga practice was an effective tool when working with students with Down syndrome, cerebral palsy, autism, sensory integration disorder, learning difficulties (Galantino et al., 2008), and attention deficit hyperactivity disorder (Balasubramaniam et al., 2012). In addition, yoga revealed a positive impact on cognitive functions in children with attention deficit and hyperactive disorder (American Psychiatric Association [APA], 2000; Harrison et al., 2004; Jensen and Kenny, 2004; Krisanaprakornkit et al., 2010; Diamond, 2012). Yoga is also associated with a positive effect on depression, anxiety, mod, self-esteem, and higher academic performance in children (Ortega et al., 2008). Additionally, yoga reduces negativity and aggression in children (Santangelo, 2012), improves social behavior in children aged 6–8 (Folleto et al., 2016), increases spatial memory scores, strategic planning, and concentration in children (Telles et al., 1993), as well as it improves cognitive performance of 7–9-year-old school children (Chaya et al., 2012).

Nevertheless, research involving yoga and Kindergarten children is relatively sparse. Therefore, the main purpose of the present study is to analyze the impact of yoga practice in comparison to generic physical education (PE) and a passive control group (CG) on (i) attention as a cognitive performance (operationalized with the visual attention test) and visual-motor coordination (operationalized with the Visuomotor precision test) and (ii) ADHD relevant behavior of inattention and hyperactivity (operationalized with the ADHD Rating Scale-IV), in 5-year-old preschool Tunisian children.

We hypothesized that in comparison to the generic PE group as well as the passive CG, the yoga group would display a significant improvement in visual attention and visual-motor precision as well as a significant reduction in inattention and hyperactivity behavior.

## Materials and Methods

### Participants

In this single-center, three-arm randomized, controlled study, 45 healthy children (28 female; 17 male; 5.2 ± 0.4 years) of a private Tunisian Kindergarten volunteered to participate. The Kindergarten is located in an urban setting and participants were from middle class families with a corresponding average to high socio-economic status. All participants and their legal guardians were informed in detail, in written form as well as orally, about the design of the study, including the potential risks and benefits. Subsequently, parents, respectively, legal guardians of the children, provided their informed written consent to participate. Participants were free to withdraw from the study at any time without further consequences. The inclusion criteria were a lack of any frequent participation in yoga exercise programs for at least 6 months prior to the study; no daily intake of medication; and for inclusion in the analysis, completion of at least 80% of the yoga sessions.

All procedures were conducted in accordance with the Code of Ethics for human experimentation of the World Medical Association, the Declaration of Helsinki (World Medical Association, 2013), as well as the ethical standards of the International Journal of Sports Medecine (Harriss and Atkinson, 2015). The experimental protocol was pre-approved by the ethical review board of the High Institute of Sport and Physical Education of University of Sfax, Tunisia.

### Overall Study Design

**FIGURE 1:**
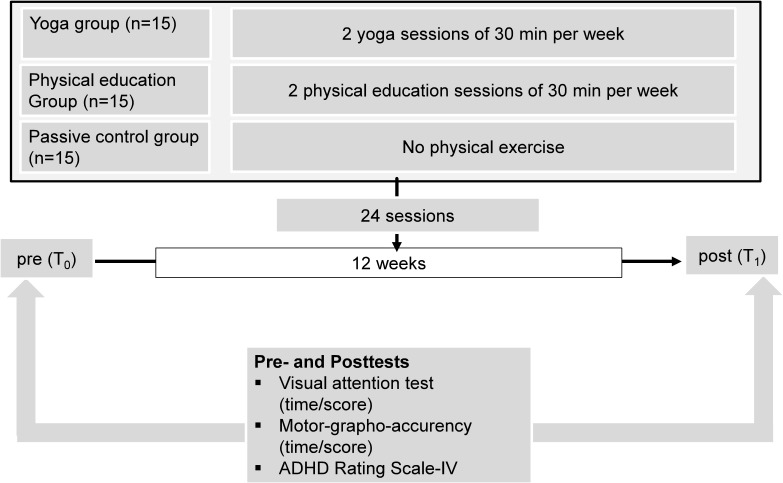
Overview of the study protocols with pre- and post-tests.

Fifteen students performed a total of 24 yoga sessions, during a period of 12 weeks (two yoga sessions per week, 30 min per session) during regular Kindergarten hours, 15 students performed a generic PE program (24 sessions, two sessions per week, 30 min per session), and 15 students served as passive CG (Figure 1). Participants were randomly assigned to one of the three groups. Prior to (*T*_0_) and after the 12 weeks of either yoga, PE, or passive control (*T*_1_), all participants completed one subtest of the Developmental Neuropsychological Assessment (NEPSY) (Korkman et al., 1998) and one subtest of NEPSY-II (Korkman et al., 2007) and the teachers evaluated the inattention and hyperactivity behaviors of the participants with the help of the school version of the ADHD Rating Scale-IV (DuPaul et al., 1998). Since Kindergarten teachers evaluated the children’s behavior with the help of the ADHD Rating Scale-IV, it was necessary to warrant impartiality of Kindergarten teachers toward the children. Therefore, Kindergarten teachers were not informed which children were assigned to the respective groups. Furthermore, teachers were not fully informed about the purpose of the study as well as about the nature and content of the two different interventions. The post-intervention testing started 72 h after the final scheduled session of yoga and PE (Figure 1).

### The Interventions

**FIGURE 2:**
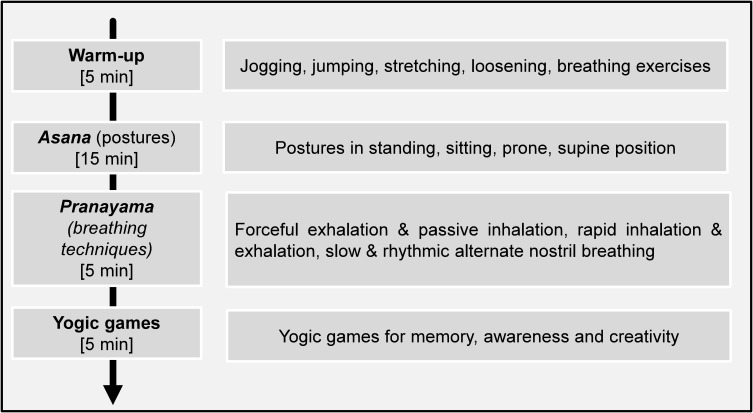
Details of the yoga program.

The yoga group performed 24 yoga sessions (two yoga sessions per week, 30 min per session) involving Hatha yoga. An adapted Hatha yoga program was applied to meet the level of the children and the goals of the research. The yoga program was conducted by a certified yoga teacher. Each session during the 12-week intervention involved a 30-min yoga program in the Kindergarten’s gym. Each session included a 5 min preparatory period as a warm-up, comprising jogging and jumping followed by yoga specific stretching and loosening exercises and breathing exercises. During the main part of yoga sessions children performed yoga postures (*Asana*) for 15 min followed by 5 min of breathing techniques. The yoga postures included postures in standing, sitting, prone, and supine position. The breathing techniques (*Pranayama*) involved voluntary regulation of breathing like breathing with forceful exhalation and passive inhalation, breathing with rapid inhalation and exhalation, as well as slow and rhythmic alternate nostril breathing. At the end of each session yogic games for memory, awareness and creativity were completed (Figure 2). Throughout the different phases of the yoga session, a story was told to motivate the children for an active participation. The level of difficulty of yoga sessions increased from week to week to match the children’s adaptations.

The PE group performed 24 regular PE sessions (two PE sessions per week, 30 min per session with moderate intensity) involving game-based activities, like basketball, soccer, handball, and relay games. The PE program was conducted by an instructor (trained undergraduate student) in the Kindergarten’s gym.

In order to blind the interventions the following procedure was applied during the intervention period: Both yoga and PE were instructed and supervised by individuals not belonging to the Kindergarten’s staff. The participating children were picked up in their respective groups by the investigators and escorted to the respective Gyms where yoga and PE were performed. The children were left in the custody of the yoga teacher, respectively, with the undergraduate student for the PE group. The children randomized to the passive CG were escorted by the investigators to other group rooms of the Kindergarten, which did not belong to the class of the children. In that group rooms, the children were free to play and engage in self-chosen activities like free play or artisanal activities. These group rooms were supervised by other Kindergarten teachers not belonging to the class of the child. All three activities (yoga, PE, and CG) were conducted parallel at the same time and in the absence of the teachers who rated the ADHD relevant behavior pretest and posttest.

Participants attended 91% of PE sessions and 88% of yoga sessions.

The CG completed no kind of physical exercise during the duration of the study.

### Visual Attention Test

The visual attention test is one out of 27 subtest of the Developmental Neuropsychological Assessment (NEPSY) (Korkman et al., 1998). An instrument that provides a comprehensive neuropsychological assessment grouped into five domains (attention/executive function, language, memory and learning, sensorimotor functions, and visuospatial processing) for children aged 3–12 years (Korkman et al., 1998). Briefly, the Visual Attention subtest is allocated to the domain Attention and Executive Functioning and comprises a two-part visual cancellation task that assesses speed and precision at detecting targets among distractors.

### Visuomotor Precision Test

The *NEPSY–Second Edition* (NEPSY-II) (Korkman et al., 2007) is an updated and modified version of the NEPSY (Korkman et al., 1998) and assessing neuropsychological functioning across the six domains attention and executive functioning, language, memory and learning, sensorimotor, social perception, and visuospatial processing with the help of several subtests. As part of the NEPSY-II, as described in detail elsewhere (Korkman et al., 2007), the subtest *Visuomotor Precision* is designed to assess neuropsychological functioning in the domain of sensorimotor function. The subtest measured the time in which participants perform a visuomotor task as well as the precision of the graphics performed in this task. The children using the preferred hand to draw lines inside of tracks as quickly and precise as possible.

### ADHD Rating Scale-IV

The ADHD Rating Scale-IV (DuPaul et al., 1998) is a norm-referenced checklist that measures the symptoms of ADHD based on the diagnostic criteria of the *Diagnostic and Statistical Manual of Mental Disorders* (American Psychiatric Association [APA], 2000). As described in detail elsewhere (DuPaul et al., 1998), the ADHD Rating Scale-IV (French version) measures the frequency of two distinct behaviors: (1) symptoms associated with inattention and (2) symptoms associated with hyperactivity/impulsivity. In the present study, the inattention and hyperactivity behaviors of the participants were evaluated by the Kindergarten teacher with the help of the school version of the ADHD Rating Scale-IV (DuPaul et al., 1998). Whereas, the same preschool teacher evaluated the child at (*T*_0_) and (*T*_1_). The teacher assesses symptom frequency using a four-point Likert scale. The questionnaire consists of 18 items, nine items relating to inattention and nine items relating to hyperactivity. The behavioral scale of the students (DuPaul et al., 1998) is an instrument for assessing frequency of inattention and hyperactivity/impulsivity behaviors. Results are described in terms of the Inattention subscale and the Hyperactivity-impulsivity subscale as well as the total score.

### Empirical Hypothesis

Yoga has a significant positive impact on the development of visual attention, visuomotor precision, and ADHD behavior in comparison to generic PE as well as in comparison to CG.

### Statistical Analysis

Statistical tests were processed using SPSS software for Windows, version 25 (IBM, Armonk, NY, United States). Mean and standard deviation (SD) values were calculated for each variable. G ^∗^power software (Faul et al., 2007) was applied to calculate the required sample size. Values for α were set at 0.05 and power at 0.80. Based on discussions between the authors, effect size was estimated to be 0.7 (medium effect). Required sample size was 55 participants. Due to practical reasons like availability, 45 children could be included.

The Shapiro-Wilk test revealed that data of *T*_0_ and *T*_1_ was normally distributed and homogeneity of variance (Levene test) was given with no transformation necessary. A repeated measures analysis of variance was performed to analyze the impact of yoga in comparison to generic PE (2 [group] × 2 [time-of-measure]) as well as in comparison to CG (2 [group] × 2 [time-of-measure]). In addition to repeated measures analysis of variance, Bonferroni–Holm corrections were performed. Furthermore, data of *T*_0_ were checked for significant differences between the three groups, applying a one-way ANOVA. A probability level of 0.05 was selected a priori as the criterion for statistical significance. To estimate practical relevance, effect sizes (partial eta squared, ηp^2^) (Richardson, 2011) were calculated with ηp^2^ ≥ 0.01 indicated small, ≥0.06 medium, and ≥0.14 large effects (Clark-Carter, 1997).

## Results

All parameters measured at *T*_0_ and *T*_1_ with accompanying statistical analyses are presented in Table 1. All parameters measured at *T*_0_ did not show significant differences between the three groups (*p* > 0.001).

**Table 1:** Parameters of NEPSY, NEPSY-II and ADHD Rating Scale-IV (means ± SD) for the control group (*n* = 15), physical education group (*n* = 15), and yoga group (*n* = 15) before (*T*_0_) and after (*T*_1_) the 12-week intervention.

		*T*_0_	*T*_1_	Time effect				Group effect				Time × group effect			
						
				*F*(1,28)	*p*	Adj. *p*	ηp^2^	*F*(1,28)	*p*	Adj. *p*	ηp^2^	*F*(1,28)	*P*	Adj. *p*	ηp^2^
Attention A [min]	Yoga	1.41 ± 0.07	1.31 ± 0.05												
	PE	1.41 ± 0.59	1.35 ± 0.05	28.38	0.000	0.007	0.503	1.80	0.191	0.025	0.060	2.62	0.117	0.050	0.086
	Control	1.41 ± 0.06	1.39 ± 0.07	12.58	0.001	0.017	0.310	8.55	0.007	0.005	0.234	5.23	0.030	0.008	0.157
Attention B [score]	Yoga	8.87 ± 1.64	12.20 ± 1.61												
	PE	8.93 ± 1.03	10.53 ± 1.30	44.47	0.000	0.004	0.614	4.86	0.036	0.008	0.148	5.49	0.026	0.007	0.164
	Control	9.33 ± 1.80	10.13 ± 1.68	30.47	0.000	0.006	0.521	2.68	0.113	0.010	0.087	11.45	0.002	0.005	0.290
VM precision A [s]	Yoga	24.58 ± 1.25	21.06 ± 1.47												
	PE	24.25 ± 1.17	23.69 ± 1.86	37.29	0.000	0.005	0.571	7.63	0.010	0.006	0.214	19.62	0.000	0.004	0.412
	Control	26.17 ± 2.09	24.64 ± 3.21	31.48	0.000	0.005	0.529	16.30	0.000	0.004	0.368	4.92	0.035	0.010	0.149
VM precision A [score]	Yoga	11.33 ± 1.45	8.93 ± 1.75												
	PE	11.73 ± 1.91	11.07 ± 1.22	15.08	0.001	0.010	0.350	8.57	0.007	0.005	0.234	4.82	0.037	0.013	0.147
	Control	11.93 ± 2.08	11.13 ± 2.45	10.97	0.003	0.025	0.282	6.92	0.014	0.006	0.198	2.74	0.109	0.025	0.089
VM precision B [min]	Yoga	1.51 ± 0.07	1.33 ± 0.08												
	PE	1.49 ± 0.07	1.46 ± 0.07	82.26	0.000	0.003	0.746	5.80	0.023	0.007	0.172	34.96	0.000	0.003	0.555
	Control	1.47 ± 0.12	1.44 ± 0.14	14.63	0.001	0.013	0.343	1.44	0.240	0.050	0.049	7.64	0.010	0.006	0.214
VM precision B [score]	Yoga	21.93 ± 2.37	18.67 ± 1.35												
	PE	21.87 ± 2.45	20.47 ± 1.56	29.22	0.000	0.006	0.511	2.23	0.147	0.013	0.074	4.68	0.039	0.017	0.143
	Control	21.93 ± 2.09	22.07 ± 2.19	6.35	0.018	0.050	0.185	17.39	0.000	0.004	0.383	7.48	0.011	0.006	0.211
Hyperactivity	Yoga	13.93 ± 1.67	9.60 ± 1.59												
	PE	13.67 ± 1.59	11.27 ± 1.28	134.22	0.000	0.003	0.827	2.11	0.157	0.017	0.070	11.07	0.002	0.005	0.283
	Control	14.47 ± 1.96	13.20 ± 1.93	58.66	0.000	0.004	0.677	14.41	0.001	0.004	0.340	17.59	0.000	0.004	0.386
Inattention	Yoga	12.40 ± 1.50	6.87 ± 1.64												
	PE	12.73 ± 1.53	10.27 ± 0.96	100.70	0.000	0.003	0.782	30.20	0.000	0.003	0.519	14.80	0.001	0.004	0.346
	Control	12.60 ± 1.99	12.73 ± 3.15	24.35	0.000	0.004	0.465	27.98	0.000	0.003	0.500	26.82	0.000	0.003	0.489
Total	Yoga	26.33 ± 2.19	16.47 ± 2.33												
	PE	26.40 ± 2.50	21.53 ± 1.55	211.56	0.000	0.003	0.883	17.66	0.000	0.003	0.387	24.37	0.000	0.003	0.465
	Control	27.07 ± 3.06	25.93 ± 4.57	52.13	0.000	0.008	0.651	33.80	0.000	0.003	0.547	32.86	0.000	0.003	0.540


**N*_*Yoga*_ = 15; *N*_*PE*_ = 15; *N*_*Control*_ = 15; min = minute; s = seconds; VM precision A (s) = visuomotor precision A; VM precision B (min) = visuomotor precision B (min); total score = total score of the ADHD Rating Scale-IV (DuPaul et al., 1998); adj. *P* = *p*-values Bonferroni–Holm corrected; PE = group physical education; CG = control group with no physical activity; F = degrees of freedom; *p* = probability; ηp^2^ = effect size partial eta-square*.

### Visual Attention

Yoga has a non-significant impact on the development of visual attention A in comparison to generic PE [time × group effect: *F*(1,28) = 2.62; *p* = 0.117; ηp^2^ = 0.086]. However, yoga has a significant positive impact on the development of visual attention A in comparison to CG [time × group effect: *F*(1,28) = 5.23; *p* = 0.030; ηp^2^ = 0.157]; yoga (*T*_0_: 1.41 ± 0.07, *T*_1_: 1.31 ± 0.05) leads to comparatively faster attention times (CG: *T*_0_: 1.41 ± 0.06, *T*_1_: 1.39 ± 0.07). In addition, yoga has a significant positive impact on the development of visual attention B in comparison to PE [time × group effect: *F*(1,28) = 5.49; *p* = 0.026; ηp^2^ = 0.164] as well as in comparison to CG [time × group effect: *F*(1,28) = 11.45; *p* = 0.002; ηp^2^ = 0.290]; in both cases, yoga (*T*_0_: 8.87 ± 1.64, *T*_1_: 12.20 ± 1.61) leads to comparatively higher scores (PE: *T*_0_: 8.93 ± 1.03, *T*_1_: 10.53 ± 1.30; CG: *T*_0_: 9.33 ± 1.80, *T*_1_: 10.13 ± 1.68). All significant interaction effects can be classified as large.

However, after Bonferroni–Holm correction, only one significant time × group interaction effect remained. Hereafter, yoga has a significant positive impact on the development of visual attention B in comparison to CG (adj. *p* = 0.005).

### Visuomotor Precision

Yoga has a significant positive impact on the development of visuomotor precision A in comparison to generic PE [time × group effect: *F*(1,28) = 19.62; *p* = 0.000; ηp^2^ = 0.412] as well as in comparison to CG [time × group effect: *F*(1,28) = 4.92; *p* = 0.035; ηp^2^ = 0.149]; in both cases, yoga (*T*_0_: 24.58 ± 1.25, *T*_1_: 21.06 ± 1.47) leads to comparatively faster visuomotor precision times (PE: 24.25 ± 1.17, *T*_1_: 23.69 ± 1.86; CG: *T*_0_: 26.17 ± 2.09, *T*_1_: 24.64 ± 3.21). Further, yoga has a significant positive impact on the development of visuomotor precision A in comparison to generic PE [time × group effect: *F*(1,28) = 4.82; *p* = 0.037; ηp^2^ = 0.147]; yoga (*T*_0_: 11.33 ± 1.45, *T*_1_: 8.93 ± 1.75) leads to comparatively lower error rates (PE: *T*_0_: 11.73 ± 1.91, *T*_1_: 11.07 ± 1.22). However, yoga has no significant impact on the development of visual attention A in comparison to CG [time × group effect: *F*(1,28) = 2.74; *p* = 0.109; ηp^2^ = 0.089]. In addition, yoga has a significant positive impact on the development of visuomotor precision B in comparison to generic PE [time × group effect: *F*(1,28) = 34.96; *p* = 0.000; ηp^2^ = 0.555] as well as in comparison to CG [time × group effect: *F*(1,28) = 7.64; *p* = 0.010; ηp^2^ = 0.214]; in both cases, yoga (*T*_0_: 1.51 ± 0.07, *T*_1_: 1.33 ± 0.08) leads to comparatively faster visuomotor precision times (PE: *T*_0_: 1.49 ± 0.07, *T*_1_: 1.46 ± 0.07; CG: *T*_0_: 1.47 ± 0.12, *T*_1_: 1.44 ± 0.14). Finally, yoga has a significant positive impact on the development of visuomotor precision B in comparison to generic PE [time × group effect: *F*(1,28) = 4.68; *p* = 0.039; ηp^2^ = 0.143] as well as in comparison to non-treatment conditions [time × group effect: *F*(1,28) = 7.48; *p* = 0.011; ηp^2^ = 0.211]; in both cases, yoga (*T*_0_: 21.93 ± 2.37, *T*_1_: 18.67 ± 1.35) leads to comparatively lower error rates (PE: *T*_0_: 21.87 ± 2.45, *T*_1_: 20.47 ± 1.56; CG: *T*_0_: 21.93 ± 2.09, *T*_1_: 22.07 ± 2.19). All significant interaction effects can be classified as large.

However, after Bonferroni–Holm correction, only two significant time × group interaction effects remained. Hereafter, yoga has a significant positive impact on both, the development of visuomotor precision A (completion time) in comparison to PE (adj. *p* = 0.004) as well as visuomotor precision B (completion time) in comparison to PE group (adj. *p* = 0.003).

### ADHD Rating Scale-IV

Yoga has a significant positive impact on the development of hyperactivity behavior in comparison to generic PE [time × group effect: *F*(1,28) = 11.07; *p* = 0.002; ηp^2^ = 0.283] as well as in comparison to CG [time × group effect: *F*(1,28) = 17.59; *p* = 0.000; ηp^2^ = 0.386]; in both cases, yoga (*T*_0_: 13.93 ± 1.67, *T*_1_: 9.60 ± 1.59) leads to comparatively lower hyperactivity behavior (PE: 13.67 ± 1.59, *T*_1_: 11.27 ± 1.28; CG: *T*_0_: 14.47 ± 1.96, *T*_1_: 13.20 ± 1.93). Further, yoga has a significant positive impact on the development of inattention behavior in comparison to generic PE [time × group effect: *F*(1,28) = 14.80; *p* = 0.001; ηp^2^ = 0.346] as well as in comparison to CG [time × group effect: *F*(1,28) = 26.82; *p* = 0.000; ηp^2^ = 0.489]; in both cases, yoga (*T*_0_: 12.40 ± 1.50, *T*_1_: 6.87 ± 1.64) leads to comparatively lower inattention scores (PE: 12.73 ± 1.53, *T*_1_: 10.27 ± 0.96; CG: *T*_0_: 12.60 ± 1.99, *T*_1_: 12.73 ± 3.15). Finally, yoga has a significant positive impact on the ADHD total score in comparison to generic PE [time × group effect: *F*(1,28) = 24.37; *p* = 0.000; ηp^2^ = 0.465] as well as in comparison to CG [time × group effect: *F*(1,28) = 32.86; *p* = 0.000; ηp^2^ = 0.540]; in both cases, yoga (*T*_0_: 26.33 ± 2.19, *T*_1_: 16.47 ± 2.33) leads to comparatively lower ADHD-total-scores (PE: 26.40 ± 2.50, *T*_1_: 21.53 ± 1.55; CG: *T*_0_: 27.07 ± 3.06, *T*_1_: 25.93 ± 4.57). All significant interaction effects can be classified as large. Following Bonferroni–Holm corrections, all reported time × group interaction effects remained significant (Table 1).

## Discussion

The aim of this study was to investigate the effects of 12-week Kindergarten-based yoga practice on visual attention, visual-motor precision, as well as on hyperactivity and inattention behavior in 5-year-old Tunisian children.

The main findings of the present study were as follows:

(1) Yoga had a significant positive effect over the 12-week intervention period on the majority of parameters of visual attention and on parameters of visual-motor precision, in comparison to the PE and CG. However, after Bonferroni–Holm correction, yoga only had a significant positive impact on the development of visual attention B in comparison to CG. Considering visual-motor precision after Bonferroni–Holm correction, yoga only had a significant positive impact on the development of visuomotor precision A (completion time) in comparison to PE, as well as on visuomotor precision B (completion time) in comparison to PE group.
(2) Hyperactivity and inattention behavior and the total score of the ADHD Rating Scale-IV improved significantly in the yoga group from *T*_0_ to *T*_1_, in comparison to the PE and CG.

The results demonstrate that children participating in the yoga program, in comparison to the PE and CG, improved their skills related to cognitive functions, measured by the subtests of NEPSY (Korkman et al., 1998) and NEPSY-II (Korkman et al., 2007), as well as their behavior in respect to inattention and hyperactivity, evaluated by the ADHD Rating Scale-IV (DuPaul et al., 1998). The amelioration of attention with the help of yoga in the present study is in line with previous results demonstrating that sensorimotor training enhances attention in children with (Banaschewski et al., 2001; Cohen et al., 2018) and without ADHD (Mak et al., 2018). Our results are also coherent with previous studies reporting that school-based prevention programs focusing on yoga or sensorimotor training for young children can reduce deficits in attention and improve academic performance (Paour and Cèbes, 2001; Chevalier and Simard, 2006; Krisanaprakornkit et al., 2010; Gothe et al., 2013). In addition, our results support findings of previous research demonstrating that yoga practice decreases ADHD symptoms in preschool children (Cohen et al., 2018). Although, no manifest ADHD diagnosis (e.g., from a clinician, psychologist, or pediatrician) was evident for participants of the present study, many participants showed symptoms of inattention, hyperactivity, and impulsivity at *T*_0_ (Table 1). With the yoga intervention, the inattention behavior in the present study was reduced effectively.

Since attention represents an important prerequisite in the processes of learning (Flessas and Lussier, 2001; McClelland et al., 2013) and is associated with better academic performance in preschool children (Duncan et al., 2007) as well as executive functions (Friedman et al., 2007) and academic performance (McClelland et al., 2013) in the adolescence, the present study demonstrated that yoga has the potential to facilitate learning processes and may contribute to academic achievement of preadolescent children. Furthermore, it seems to be important to work on attention problems at an early age, because attention problems during childhood seem to be associated with poorer executive functions in late adolescence (Friedman et al., 2007). Recent reviews calculated a positive small to moderate effect on attention for acute physical activity and a large positive effect on attention for chronic physical activity (de Greeff et al., 2018). In agreement with the recent review (de Greeff et al., 2018), the present study revealed large positive effect sizes for the chronic yoga intervention on attention. It is suggested that the positive effects on attention leading to increased time children are engaged in academic tasks, which has the potential of better academic performance in the long run (Duncan et al., 2007; Oberer et al., 2018). It is hypothesized that both increased physical activity, respectively, an increased physical fitness as a result of the latter leading to an increased activity in selective structures of the brain, causing functional and structural connectivity (Esteban-Cornejo et al., 2017). As a result, physical activity potentially facilitates factors of cognitive performance and executive functions (Diamond, 2000; Colcombe and Kramer, 2003). For the exact underlying mechanisms of structural and functional brain changes associated with physical exercise in children, the available research is very limited in number and scope. Whereas, associations of cardiorespiratory fitness and speed-agility in obese children (8–11 years) with greater gray matter volumes, which in turn reflect children’s academic performance, could be demonstrated recently (Esteban-Cornejo et al., 2017). Additionally, an 8-month aerobic exercise program with 8–11-year-old obese children caused improved white matter integrity in the brain tract connecting frontal and temporal lobes (Schaeffer et al., 2014). In addition, an improved memory performance in children was associated with improved fronto-temporal circuitry as a result of aerobic exercise (Erickson et al., 2011). To the best of our knowledge, no studies are available on yoga and the effects of structural alterations of brain matter in children. Whereas, reviewed data suggest several structural alterations and activation changes of brain structures (e.g., amygdala, frontal lobes, white matter) in adults as a result of yoga (for detailed description of specific changes, see Desai et al., 2015). Similar alterations in structural and functional capacities in the brain of children may be responsible for the alterations of attention and behavior in the present study.

The present results imply that improvements in cognitive tasks were achieved after a yoga intervention compared to generic PE and the usual Kindergarten activities as represented in the CG. These results are in agreement with previous studies, which reported that young yoga participants showed significant differences over time on measures of executive functions (Manjunath and Telles, 2004; Chaya et al., 2012; Purohit and Pradhan, 2016). Likewise, Telles et al. (1993) revealed that children, practicing yoga for 10 days, improved in spatial memory scores, strategic planning, and concentration. In the same context, a significant improvement following 12 weeks of school-based yoga was observed for cognitive functions and memory in 13-year-old children (Verma et al., 2014). In agreement with studies on yoga with older children, our data demonstrate that preschool-based yoga is a suitable physical exercise to support cognitive abilities of preschool children. Since the level of physical activity, executive functions, and visual-motor coordination in Kindergarten children seem to be associated with later academic achievement (Oberer et al., 2018), it seems to be beneficial to implement regular yoga classes at preschools to contribute to the basis for academic achievement in young children. In this context, several authors advocate the potential of yoga for schools as an enjoyable and cost-effective exercise to improve cognitive and behavioral skills, which are relevant for school and may contribute to academic achievement (Kauts and Sharma, 2009; Chaya et al., 2012; Verma et al., 2014). The observation of the practical application of yoga at schools worldwide reveals that yoga is increasingly important in the school setting (Flak, 2003; Chaya et al., 2012) since it is recognized in the curriculum in France, Brazil, Canada, and Italy (Flak, 2003). Additionally, in Italy, classroom-based yoga is performed in all schools since 2000 (Flak, 2003).

Although no explicit ADHD patients were among participants of the present study, the yoga intervention reduced the behavior of hyperactivity and inattention significantly and to a practical relevant extent, which is in line with numerous studies. Previous research demonstrated that a sensorimotor training program for children leads to the reduction of behavioral problems (Eggert and Lütje, 1991) and ameliorates anger control and anxiety (Khalsa et al., 2012), factors that potentially interfere with academic achievement and psychosocial well-being. Furthermore, classroom-based yoga leads to a significant improvement in emotional and psychosocial quality of life and reduced anxiety in third-grade students (Bazzano et al., 2018). Additionally, Noggle et al. (2012) reported that school-based yoga in high school students caused increases in measures of psychosocial well-being in comparison with the regular PE. Consequently, yoga could be considered as a method to teach coping skills for young students which potentially contribute to mental health and psychosocial well-being.

As noted by Rossner (1995), yoga would allow relaxation to improve attention to task and spatial relationships in the environment, which we confirmed by our results. The findings of the present study provide evidence for the importance of practicing yoga at Kindergarten as it helps reach higher cognitive performance and consequently potentially higher academics performances in children. Thus, yoga seems to be an appropriate tool to a cognitive education to preschool Tunisian children as a complement to the usual social, emotional, and artisanal learning activities. As recommended (Paour and Cèbes, 2001), yoga would allow the preschool children to effectively engage attentional abilities and improve the executive self-regulation of the action.

### Limitations

Whereas, it would be useful to have a larger sample since our study consisted of 45 participants and this may present a limitation since the variability may be significant in this sample. Furthermore, a greater number of participants would have given more statistical power to the data interpretation with less risk of calculating type 2 errors. At the same time, the small sample size allowed us to increase availability, motivation, and compliance of children during participation.

The duration of a yoga session is usually approximately 1:20 h (Lark, 2003) and the 30 min of yoga allotted to each class in the present study could represent a limitation. We reduced the sessions to 30 min in order to respect the attention span of the children as well as the schedule allocated for the daily routines of the Kindergarten. Nevertheless, the duration of 30 min of yoga sessions in the present study is within the usual range of 30–40 min of comparable studies (Chou and Huang, 2017; Bazzano et al., 2018).

The inattention and hyperactivity behaviors of the participants were evaluated by the Kindergarten teacher with the help of the ADHD Rating Scale-IV (DuPaul et al., 1998). The Kindergarten teachers were not informed about the hypothesis of the study and blinded to the intervention groups and the passive CG. We only informed Kindergarten teachers that different kinds of physical exercise were tested on cognitive functions in the children. Whereas, we cannot exclude that children told their Kindergarten teachers about the performed intervention or that Kindergarten teachers eventually had intuitions concerning expected results of the yoga intervention.

The present study is part of a research project focusing on graphic design and its link with motor and cognitive aspects. We consider fine motor skills as especially important during preschool age since writing is a fine motor activity that is initiated during the preschool period and requires perceptual-motor skills. In children aged between 2–4 years, the vision serves as a primary guide for graphic activities like writing (James, 2010; Maldarelli et al., 2015). Therefore, we did not include auditory attention tests. Since auditory stimuli are an additional prerequisite for graphic activities, we are looking forward to implement auditory attention tests in the following parts of the research project.

## Conclusion

Twelve weeks of two 30-min yoga sessions per week improved certain parameters of attention, visual-motor precision, and reduced behavior of inattention and hyperactivity in 5-year-old Kindergarten children. The yoga program improves attention, visual-motor precision, and behavior to a higher extent compared to generic PE and the usual Kindergarten activities as represented in the passive CG. As a consequence, Kindergarten-based yoga classes represents a sufficient and cost-benefit effective exercise activity which may enhance functions relevant for learning among young children and could be added as a complement to social, emotional, and artisanal learning activities at the Kindergarten.

## Ethics Statement

In this single-center, three-arm randomized, controlled study, 45 healthy children (28 female; 17 male; 5.2 ± 0.4 years) of a private Tunisian Kindergarten volunteered to participate. All participants and their legal guardians were informed in detail, in written form as well as orally, about the design of the study, including the potential risks and benefits, before providing their informed written consent to participate. Participants were free to withdraw from the study at any time without further consequences. The inclusion criteria were a lack of any frequent participation in yoga exercise programs for at least 6 months prior to the study; no daily intake of medication; and for inclusion in the analysis, completion of at least 80% of the yoga sessions.

All procedures were conducted in accordance with the Code of Ethics for human experimentation of the World Medical Association, the Declaration of Helsinki (World Medical Association, 2013), as well as the ethical standards of the International Journal of Sports Medecine (Harriss and Atkinson, 2015). The experimental protocol was pre-approved by the ethical review board of the High Institute of Sport and Physical Education of University of Sfax, Tunisia.

## Author Contributions

All authors have made a substantial, direct, and intellectual contribution to the work and approved it for publication.

## Conflict of Interest Statement

The authors declare that the research was conducted in the absence of any commercial or financial relationships that could be construed as a potential conflict of interest.
